# On pethidine: The ‘accidental’ opioid that became a ‘model’ analgesic agent

**DOI:** 10.1177/0310057X261442548

**Published:** 2026-06-14

**Authors:** Laurence E Mather, Geoffrey T Tucker

**Affiliations:** 1 Department of Anaesthesia, The University of Sydney, Sydney, NSW, Australia; 2 Department of Clinical Pharmacology, University of Sheffield, Sheffield, South Yorkshire, UK

**Keywords:** Analgesia, history, meperidine, pethidine, pharmacokinetics, routes of administration, toxicity

## Abstract

A 1939 German publication on a search for novel synthetic atropine substitutes reported that ‘Substanz III’, named Dolantin (now generically known as pethidine and meperidine), was found coincidentally to also have morphine-like, albeit markedly weaker, analgesic properties. With a chemical structure not obviously related to morphine, it was thus an ‘accidental opioid’ and was soon successfully marketed in many countries for pain relief. By the 1970s, continuing advances in analytical chemistry were enabling pharmacokinetic studies of countless drugs. Among opioid analgesic agents, pethidine’s larger dosing requirements made it suitable for such research, which was performed in healthy volunteers and patients with various doses and routes of administration, with some reports also including pharmacological responses. Standard postoperative intramuscular pethidine administration revealed steep and unpredictable plasma concentration–analgesic response relationships characterised by marked variability between patients. Novel computer-designed intravenous infusions based on typical pethidine pharmacokinetic data were trialled to replace intramuscular injections. These infusions improved the reliability of postoperative pain management but could not overcome the variability in individual patient requirements. This limitation accelerated the development of empirical ‘patient-controlled analgesia’ techniques, initially with pethidine, and subsequently with other opioid analgesic agents, thereby enabling personalised pain management. The 1970s’ discovery of spinal opioid receptors and mechanisms led to neuraxial pethidine administration being investigated and subsequently integrated into standard clinical practice. Although pethidine has now been superseded in many countries, it served as an important ‘model’ drug to investigate how to improve the use of opioid analgesic agents for the management of severe pain.

## Introduction

This essay discusses some pertinent aspects of the history and pharmacology of pethidine. Both the academic literature and lay press are replete with reports and articles about pethidine. This essay therefore presents a selection from two seminal periods: that of its discovery and introduction in the 1930s and 1940s, and that of the pharmacokinetic–pharmacodynamic research in the 1970s and 1980s.

## Pethidine: the ‘accidental opioid’ and ‘model’ analgesic agent

The German chemist Otto Eisleb (1887–1948) and pharmacologist Otto Schaumann (1891–1977) published a brief research paper in 1939 about their research to produce a novel synthetic atropine-like spasmolytic agent.^1^ Their ‘Substanz III’, named Dolantin (Figure 1), had been found, by chance, to have morphine-like, albeit much weaker, analgesic activity. With its chemical structure not being obviously related to morphine and its congeners, this substance, pethidine, was thus an ‘accidental opioid’.

**Figure 1.**
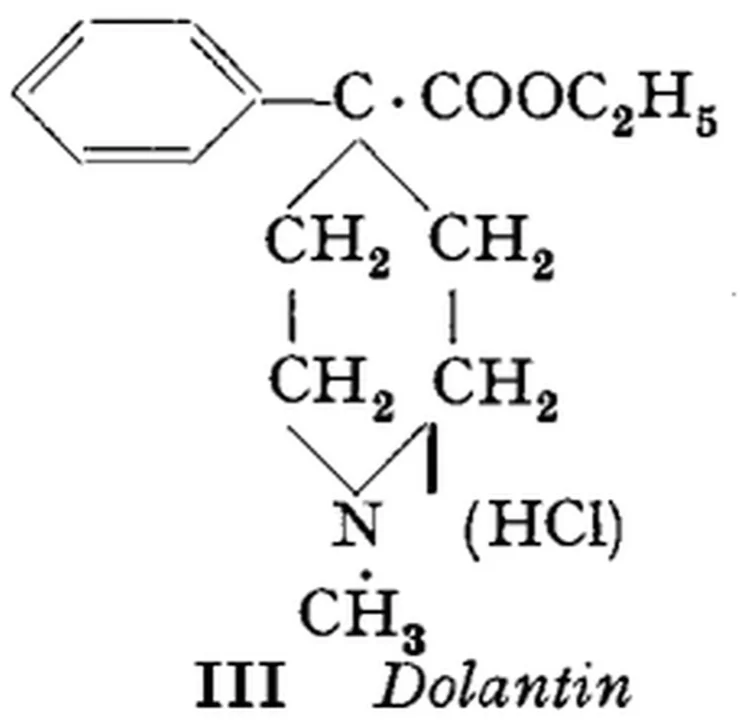
The structural formula of ‘Substanz III’, Dolantin (pethidine), as given by Eisleb and Schaumann (1939).^1^ Reproduced with permission of the publisher. *Note*. The black vertical bar (lower right) is not a part of the structural formula—its origin is unclear.

By the early 1940s pethidine had been widely accepted into clinical practice as an analgesic agent, also having potentially advantageous spasmolytic and sedative actions. By being markedly less potent than its contemporary opioid analgesic agents, the larger clinical doses required of pethidine generated substantially higher biofluid concentrations than of those agents. By the early 1970s these concentrations were reliably measurable with gas-liquid chromatography (GLC), which was becoming widely used in analytical chemistry laboratories. This led to the time course of pethidine blood, plasma and urine concentrations being determined from different modes of administration in patients and in healthy volunteer subjects, sometimes accompanied by reports of its pharmacological effects. Analytical techniques for measuring the biofluid concentrations of the other foremost opioid analgesic agents were being developed concurrently but were not yet sufficiently specific (e.g. morphine) and/or sufficiently sensitive (e.g. fentanyl) for comparable research. Thus, the pharmacokinetic and pharmacodynamic data generated enabled pethidine to become useful as a primary ‘model’ analgesic agent for further research into opioid-based pain management.

Pethidine (‘International Non-proprietary Name’) is also commonly known as meperidine (‘United States Adopted Name’), as well as several other non-proprietary and many brand names. Its chemical name, 1-methyl-4-phenyl-4-piperidinecarboxylic acid ethyl ester, conveys two of its most important chemical properties: a tertiary amine (piperidine) and an ester. Both are relevant to its principal metabolic pathways: N-demethylation to norpethidine (normeperidine) and ester hydrolysis to pethidinic (meperidinic) acid. Norpethidine-induced central nervous system (CNS) toxicity would subsequently become responsible for the withdrawal of pethidine in many countries. Nevertheless, a cursory search by way of Google Scholar of publications in 2024 containing the keywords ‘pethidine OR meperidine’ ‘excluding citations’ retrieved about 3,300 results, so it is clear that pethidine is still of substantial interest.

## Pethidine: the backstory

By the 1930s, much commercial drug development had evolved into a system of synthesis and testing of new derivatives and analogues of natural compounds of known pharmacological activity. Promising new compounds would then undergo testing for their pharmacological activity against standard substances in a variety of *in vivo* and *in vitro* bioassays. Pethidine came from this system in 1932.^2^

In their 1939 paper, Eisleb and Schaumann reported,

With the discovery of the structure of atropine as a tropic acid ester of the basic alcohol tropine, attempts were made to synthesize similarly constructed compounds. The same principle was followed here that had already led to such great success with cocaine, by varying both the acid content and the basic alcohol of atropine . . . It was now interesting to investigate how a measure that had already been successfully used in synthetic local anesthetics would affect the atropine series, namely the transfer of the basic group from the alcoholic part of the molecule to the acid part.^1^

The ‘basic group’ referred to the amine functional group, and three chemically related amino-ester compounds with spasmolytic properties were described in the paper. Of these, their Dolantin has the amine group incorporated into the cyclic structure of a substituted piperidine, which is also part of the structure of morphine and many later synthetic opioid analgesic agents.

From their tests of Dolantin in white mice, the authors stated that,

the subcutaneous injection of 1/5 of the lethal dose leads to a complete elimination of the sensation of pain. This central analgesia is not accompanied by any narcotic symptoms, but rather by a slight increase in excitement. As a visible sign of this central disinhibition, the animals show the tail phenomenon that is characteristic of morphine: the animals carry their tails in a peculiarly rigid position, bent in an S shape over their backs.^1^

—a critical observation! This ‘visible sign’ became commonly known as the ‘Straub tail effect’. It came from a brief report in 1911, in which German pharmacologist Walther Straub (1874–1944) stated,

If a small amount of morphine is injected under the skin on the back of white mice, their tails go into a catatonic state, which manifests itself in the fact that they lie almost parallel to the spine in extreme dorsal flexion . . . The reaction is specific to morphine and does not occur with other alkaloids.^3^

Eisleb and Schaumann concluded ‘This compound [‘Substanz III’, Dolantin, pethidine] was the first to combine the spasmolytic properties of atropine and papaverine with an analgesic effect similar to that of morphine.’^1^ Pethidine was thus an ‘accidental opioid’.

An application for a German patent (English) titled ‘Process for the preparation of piperidine compounds’ (i.e. including pethidine-like molecules) was filed on 8 August, 1937 by Eisleb as inventor, and assigned to IG Farbenindustrie AG,^4^ the large German chemical conglomerate that also included Farbwerke Hoechst, where Eisleb was working, and Bayer AG. The patent was granted on 3 August, 1939, then marketed by Bayer as Dolantin, an analgesic agent having spasmolytic properties. On 4 August, 1938, Eisleb filed US Patent 2,167,351 titled ‘Piperidine compounds and a process for preparing them’, assigned to the Winthrop Chemical Company in New York. The US patent, giving detailed methods for their synthesis, was granted on 25 July, 1939,^5^ from which pethidine was introduced into the USA, followed by marketing with the brand name Demerol. From the beginning, pethidine was commonly referred to by its local brand name. In Germany, it was marketed as Dolantin, having initially been named Eudolat but then changed to Dolantin because of the similarity to Eukodal (oxycodone). In the UK it was named Dolantal.

In 1940, Schaumann wrote a more comprehensive medicinal chemistry-oriented paper (English) titled ‘On a new class of compounds with spasmolytic and central analgesic efficacy, with special reference to 1-ethyl-4-phenyl-piperidine-4-carboxylic acid ethyl ester (Dolantin)’.^6^ In it, Schaumann elaborated on the preclinical pharmacological properties of a range of pethidine-like substances in laboratory animal tests, with the emphasis on understanding the effects of small chemical modifications on pharmacological activity, including comparative tests of pethidine with morphine for analgesia and systemic toxicity. The summary reported that pethidine had

a central analgesic effect of morphine-like character, whereby the narcotic component of the morphine effect takes a back seat . . . Morphine is therefore not to be regarded as a phenanthrene or isoquinoline derivative, but rather as a derivative of 4-phenyl-piperidine . . . It is suggested that the narcotic effect of morphine, which is lacking in the new compounds, is linked to the grouping of a substituted phenylethylamine contained in this and other drugs.^6^

This paper clarified the essential common structural chemical features of the synthetic and phytochemical substances, and their pharmacology. It was also the subject of a more recent biographical sketch of Schaumann along with an historical review of the key elements of the pharmacology leading to the clinical introduction of pethidine and the importance of the phenyl-piperidine group in the structure of other opioid analgesics.^2^ Schaumann later wrote reviews describing further developments in synthetic morphine-like analgesic agents, including methadone and ketobemidone,^7^ as well as potency differences between the stereoisomers of methadone.^8^

Many preclinical and clinical studies of pethidine soon followed its introduction, principally investigating its three main actions: analgesia, spasmolysis, sedation.^9–15^ With World War II already happening in Europe and looming in the USA, and with the increased wartime demand for potent analgesics, this was timely.^12^ Moreover, the need for an assured local supply of a versatile analgesic agent, not dependent on imported phytochemical sources, was imperative. It is also intriguing that pethidine knowledge rapidly became available across adversarial countries, presumably for commercial reasons.

In his 1944 doctoral thesis ‘Demerol, new substitute for morphine’, Carl Arthur Walvoord presented a literature survey with 31 references of preclinical and clinical studies of pethidine, concluding that it

possessed the following clinical advantages over morphine: (1) spasmolytic activity for cases where morphine is pharmacologically contraindicated, (2) rapid dissipation of effects beneficial in the presence of respiratory depression or urinary retention, (3) although prolonged use may lead to habituation, it appears to have less liability than morphine, (4) may be used in patients with severe anaemia, disease of liver or kidneys or bronchial asthma, (5) satisfactory as obstetric analgesic, and (6) useful as a pre-anaesthetic analgesic having fewer unfavourable side effects than morphine.^16^

Nonetheless, the morphine-like addicting properties of pethidine were quickly recognised, first in Germany,^17,18^ and then elsewhere.^19^ The US congressional legislative process hurriedly amended the federal narcotic laws to include this new agent, where it was listed by yet another generic name, isonipecaine.^20^ This name also reflects its chemistry, where pethidine can be viewed as a derivative of isonipecotic acid (piperidine-4-carboxylic acid), with the suffix ‘ine’ specifically designating an amine. Interestingly, the ‘caine’ suffix also foreshadowed discovery of its local anaesthetic properties.^21^ A decade earlier, Schaumann had worked on the pharmacology of novel synthetic local anaesthetic agents, leading to the introduction of ‘Pantokain’, the German original brand name for amethocaine (tetracaine in the USA), the synthetic prototypic long-acting local anaesthetic agent.^22^

By the early 1940s, pethidine use had become ubiquitous in medical practice as a morphine substitute. Advertisements were included in prominent medical journals: in a full-page advertisement placed by the Burroughs Wellcome Company of London for its trade brands for pethidine tablets (‘Tabloid’) and injectables (‘Hypoloid’), it was stated that it ‘may be used for pre- or postoperative medication to produce obstetric analgesia, to control the pain associated with malignant growths, cardio-vascular and neurological conditions, and to relieve dysmenorrhea and biliary renal and intestinal colic . . . is well tolerated in therapeutic doses, and has little habit-forming tendency.’^23^

Moreover, pethidine had become important enough to be mentioned in wartime newspapers. An English newspaper, for example, carried the headline ‘A PAIN KILLER’, then continued ‘It is good news, especially for our Service men and women to read that a new drug has been concocted . . . It is called pethidine’.^24^ Others picked up on the dependence issues, one carrying the striking headline ‘GERMAN DRUG Kills Pain—Becomes Habit’.^25^ The US press also picked up on the dependence and the ensuing legal consequences: one with the headline ‘Narcotics Law Control Sought for Synthetic Product’, led with the paragraph ‘The synthetic substitute for morphine developed in Germany to relieve the pain of wounded soldiers’, and went on to discuss how concerns about its illicit use and lack of rigid controls were bringing it to the attention of the law, and quoted the well-known (Federal Bureau of Narcotics chief) Harry Anslinger as saying ‘it is a dangerous habit-forming drug, which should be placed under the same rigid controls as morphine.’^26^ An Australian newspaper reported that ‘world research is feverishly seeking synthetic substitutes for morphine, active principle of the opium poppy . . . . Enter pethidine, alias dolantin, alias demerol’ Then, after a long description of its pharmacological properties and medical uses, the article continued, ‘This rather technical account may bore lay readers, but there are vast international and sociological interests involved’.^27^

By the 1960s, typical anaesthesia and pharmacology textbook chapters on ‘narcotic analgesic agents’ routinely commenced with a discourse on morphine and its phytochemical congeners such as codeine and semi-synthetic derivatives such as diacetylmorphine (now commonly known as heroin, the original trade name of Bayer), and then some synthetic morphine relatives, typically relating that pethidine was the first, followed by methadone, ketobemidone and newer ones such as fentanyl, before concluding with a section on morphine antagonists. The pethidine narrative would usually cite the original Eisleb and Schaumann paper,^1^ mentioning its emergence from the quest for a novel synthetic atropine-like antispasmodic agent, unexpectedly finding the mouse ‘Straub tail effect’,^3^ and thereby establishing its analgesic properties. Broader chapters might mention a morphine ‘receptor’ then being hypothesised on the basis of the common stereochemical shape among the morphine-like analgesics and antagonists.^28^ From the 1970s, such chapters would include the many new synthetic agents, and would have progressed from referring to ‘narcotic’ analgesic agents to ‘opiates’, and later, to ‘opioids’. The more scientifically oriented chapters would include a narrative on then-emerging research on opioid receptor subtypes, endogenous opioids, and receptor mechanisms.^29,30^ Contemporary chapters are more likely to commence with opioid receptors and mechanisms before leading on to particular analgesic agents.^31^

## Pethidine: becoming a pharmacokinetic model

In the 1960s, pethidine was commonly being used for premedication before surgery and for the treatment of severe pain, particularly after surgery and in childbirth. Concurrently, pharmacokinetics, essentially the quantitative study of the influence of the body on a drug through measurements of its time course in biofluids (usually blood and/or plasma and/or urine), increasingly often through reference to mathematical models, was becoming established as a new subdiscipline of clinical pharmacology.^32^ Such studies required reliable methods to measure the biofluid drug concentrations from relevant doses. For pethidine, the spectrophotometric methods developed in the 1940s^33–35^ were not sufficiently sensitive for this kind of research, nor could they differentiate pethidine from its metabolite norpethidine, or from unrelated amine-containing drugs such as lidocaine (lignocaine) if used concurrently, and this was not acceptable. Throughout the 1960s, GLC had been developing as a technique for separating and measuring substances in a mixture and was ideal for measuring biofluid concentrations of many drugs.^36,37^

By the 1970s, sufficiently sensitive and specific GLC methods had been developed for measuring the concentrations of pethidine after normal clinical doses.^38–41^ Ensuing studies of pethidine determined the time course of its blood and/or plasma concentrations after intravenous,^42–45^ intramuscular^46–48^ and oral^49,50^ routes of administration in healthy volunteers and patients, as well as its placental transmission.^51^ These studies determined its relevant pharmacokinetic properties, including distribution, clearance, absorption rate and bioavailability, typically by reference to standard compartment pharmacokinetic models.^52^

Some of the studies in healthy volunteers also included attempts to relate pethidine plasma concentrations to the subjective pharmacological effects generated, as might occur during postoperative pain management. One such study reported that the onset of a range of subjective effects, including concentration difficulties, mouth dryness, visual disturbances, drowsiness and speech slurring occurred when the circulating pethidine plasma concentrations exceeded approximately 0.3 mg/L, and had dissipated when pethidine concentration had decreased to approximately 0.15 mg/L (Figure 2).^48^

**Figure 2.**
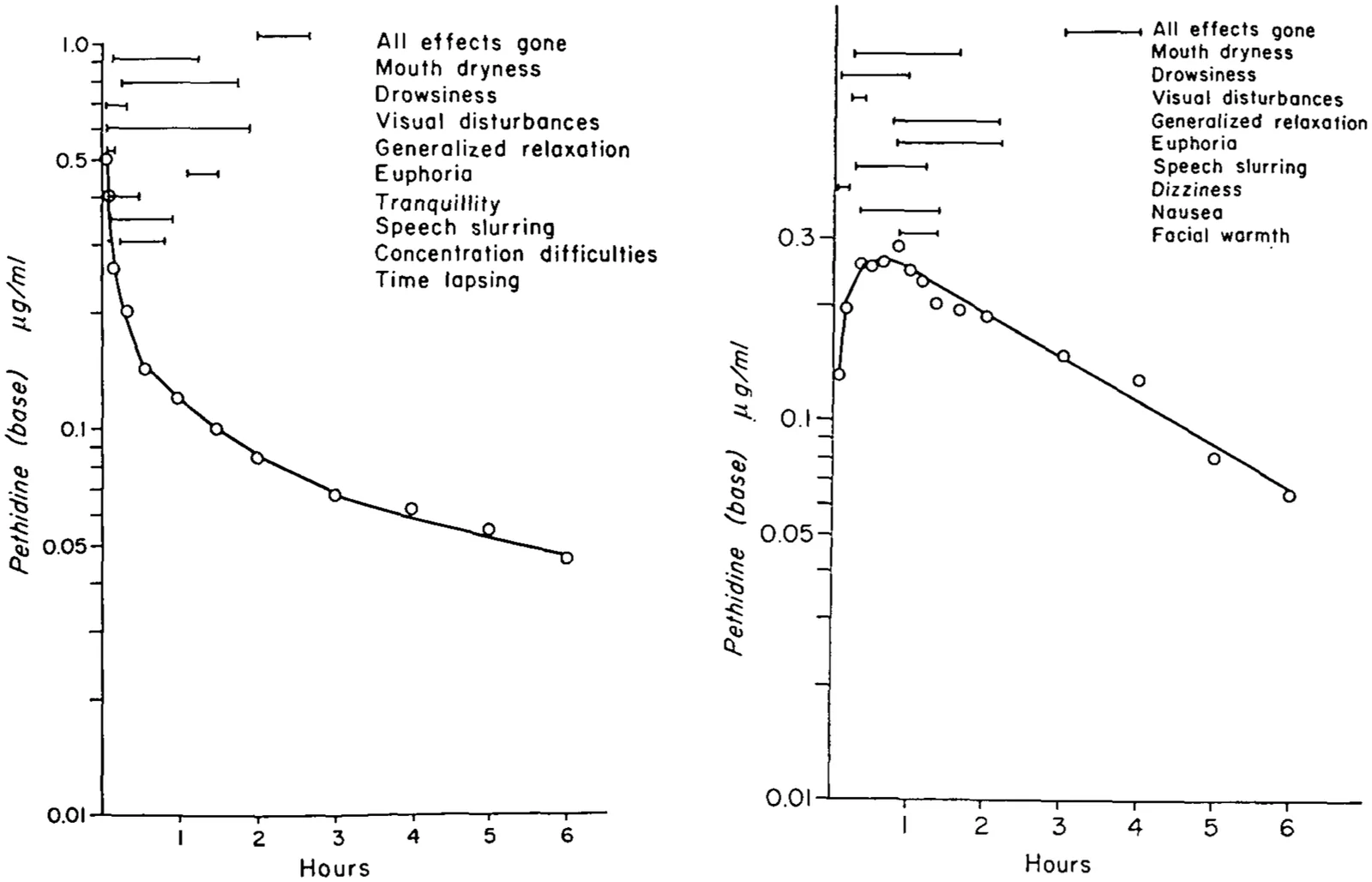
Early attempts to associate subjective effects of pethidine with its plasma concentrations in healthy unpremedicated volunteer subjects: Left: after 50 mg intravenous injection (over 1 min); Right: after 100 mg intramuscular (gluteal) injection.^48^ Reproduced with permission of the publisher.

Studies performed in surgical patients having pethidine premedication also noted abrupt increases of pethidine plasma concentrations coinciding with the induction of general (halothane) anaesthesia.^43,48^ This finding was investigated in a large animal model and found to be caused by general anaesthesia-induced haemodynamic perturbations that affected pethidine tissue distribution and elimination processes,^53^ and that these effects were a common effect of general anaesthesia on many drugs.^54^ Other studies reported on pethidine pharmacokinetics under special conditions such as pregnancy or pathology, and some studies also included measurement of the extravascular distribution of pethidine and/or its metabolites.^55–61^

The common objective of such studies was to describe the behaviour of the drug in the body and thereby provide a rationale for its better use in patient management. From a review of numerous pethidine pharmacokinetic papers produced during the 1970s, it was concluded ‘Correlations between plasma pethidine concentrations and side effects such as respiratory depression, have been established, but considerable overlap exists between concentrations producing therapeutic and non-therapeutic effects . . . The current practice of intermittent pethidine administration . . . results in fluctuations in pethidine plasma concentrations which are associated with incomplete pain relief and side effects’.^62^

Continuing studies in surgical patients found that the peak pethidine plasma concentrations from the same intramuscular dose could vary around two-fold within patients, but nearly five-fold between patients, with the times to reaching those peaks varying up to three-fold and seven-fold, respectively.^63^ Additionally, the pethidine plasma concentration–analgesic response relationships for individual patients were found to be very steep, with the minimum effective (plasma) analgesic concentration (MEAC) that was associated with a patient’s report of complete analgesia varying unpredictably, around four-fold between patients (Figure 3).^63,64^ Another review in the early 1980s included the more recent pethidine pharmacokinetic studies, along with clinically acquired data on the pain relief provided. The authors concluded that, ‘Dosage regimens of pethidine which produce variable or fluctuating blood concentrations can be expected to achieve inconsistent relief of pain. Therefore, the use of continuous intravenous infusions of pethidine appears to be a rational new approach to acute pain management’.^65^

**Figure 3.**
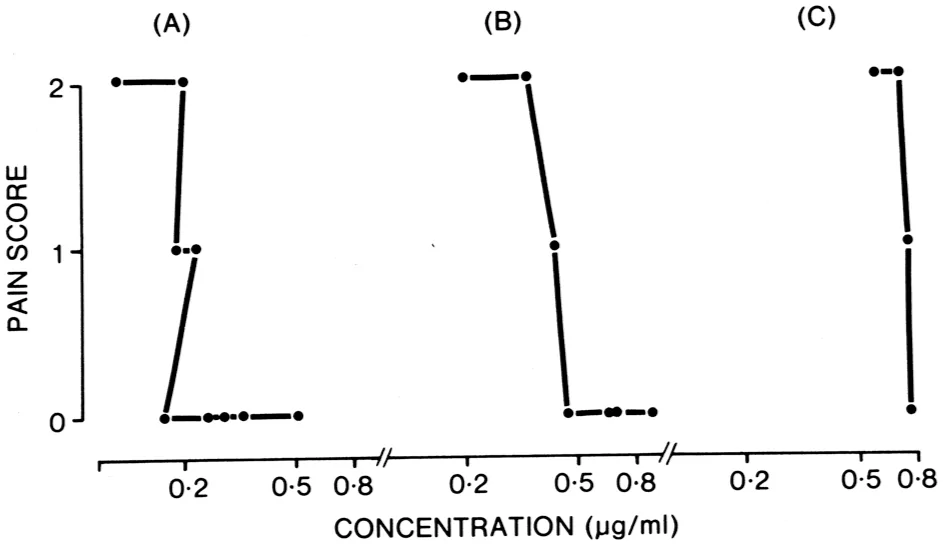
Blood pethidine concentration–response curves for three individual adult patients receiving intramuscular pethidine for postoperative pain management. Following surgery, each patient received 75 mg pethidine (as HCl) injection into the buttock, followed by 100 mg as required for 20 h, then 75 mg as required. Pain scores were recorded hourly by an independent research nurse on the basis of the answer to the question ‘Do you have pain now?’. If none, then a score of 0 was recorded; if moderate, but the patient was acceptably comfortable, then a score of 1 was recorded; if severe, then a score of 2 was recorded. Follow-up questions were asked to determine the nature and site of the pain, to confirm that the pain was originating from the surgical site.^64^ Reproduced with permission of the publisher.

Because of its more complex chemistry, morphine was not amenable to the same GLC methodology, and required chemical derivatisation to facilitate its volatility required for analysis. It was not an easy procedure for a routine analytical method. The development and application of a radioimmunoassay (RIA) procedure for morphine in 1970^66^ was therefore a noteworthy achievement. However, unlike GLC, RIA requires specialised laboratory equipment for radioactivity handling and disposal, as well as expensive radio isotope-counting technology, and was therefore less available to researchers. Moreover, the early RIA methodology had problems in that it lacked the required specificity. Spector and Vessell, in the first report of using the RIA method, recorded that ‘This method measures morphine and some of its metabolites’.^67^ Laitinen and colleagues stated that ‘Our (Spector) method measures both free and conjugated morphine and probably the N-demethylated metabolites also . . . For these reasons it is understandable that no relationship was observed between plasma morphine and pain relief’.^68^ While other morphine RIA variants were developed, it was not until the late 1970s that methods had been developed to allow comparisons to be made between the various assay techniques.^69–71^ Although some research groups performed extractions to remove the polar glucuronide metabolites before measurement, the problem of normorphine cross-reactivity persisted. Interestingly, others later noted that the minimum analgesic plasma concentration of morphine determined by gas chromatography was approximately one-half of that previously determined by RIA.^72^ The principal morphine metabolites have pharmacological activity^73^ and, unsurprisingly, the ratios of the metabolites to morphine also vary among individuals.^74^

The RIA for morphine led to commercial RIA kits being developed for morphine and later for fentanyl (as well as some other drugs). However, it was not until the late 1970s and into the 1980s that morphine pharmacokinetics could be studied with GLC and rapidly emerging high-performance liquid chromatography (HPLC) methodology with the same facility that pethidine had been studied some years earlier, resulting in comparable quality data.^72,75^ Interestingly, an RIA method was also produced for pethidine, but it had problems of cross-reactivity with norpethidine and could not compete with the relative simplicity of the GLC pethidine assay.^76^

The fentanyl story is similar, but was further hampered by the sensitivity in measuring its much smaller concentrations. A 1977 novel RIA method for fentanyl^77^ was reported to be specific and was quickly adopted for clinical studies, but it was more pertinent to studies of higher fentanyl doses. It was generally acknowledged that the lack of a routine, specific assay was a problem for studying the time course of analgesic fentanyl plasma concentrations.^78–80^ In the late 1980s, long after pethidine became that ‘model’ analgesic agent, developments especially in mass spectrometry (for GLC and HPLC) and electrochemical cells (for HPLC) could give the required detector selectivity and sensitivity for reliably measuring biofluid analgesic concentrations of morphine, fentanyl and most other opioids. As these became more widely used, greater insight could be provided into the successes and failures in patient pain management.^81^

## Pethidine: becoming a model analgesic for improving pain management

In the 1970s, leading journals were publishing commentaries about the poor management of postoperative pain, using such terms as ‘It is an indictment of modern medicine that an apparently simple problem such as relief of postoperative pain remains largely unsolved’,^82^ and that ‘the problem awaits a radical new approach’.^83^ A ‘radical new approach’ was emerging.

The first ‘new’ approach was to test the efficacy of eliminating the variability inherent in the intramuscular route of administration. Aided by then-new computer modelling techniques, the previously derived pharmacokinetic data for pethidine were used to design a three-stage intravenous infusion regimen to rapidly achieve and then provide stable postoperative analgesia by producing pethidine plasma concentrations slightly in excess of the MEAC. The infusion regimen was administered by a syringe pump with the same total pethidine dosage as used in the ‘conventional’ intramuscular approach.^84^ The infusion approach was apparently ‘radical’ and ‘new’ enough to attract lay press attention, when an Australian newspaper headlined ‘They’ve broken the pain barrier’, accompanied by explanations and illustrations of the ‘new’ intravenous infusion and ‘conventional’ intramuscular approaches.^85^

This intravenous infusion approach was generally successful for the majority of patients but, being based on average pharmacokinetic and MEAC values, the inter-patient variability problems remained. Attempts to individualise the regimen were not successful with the available systems. Moreover, it was not possible to predict an individual patient’s pethidine blood concentrations from elementary anthropometric variables, even when including their hepatic blood flow estimated from indocyanine green clearance, and it was not possible to predict an individual patient’s MEAC from elementary psychometric testing.^86^

The second ‘new’ approach came with individualised dosing in the form commonly referred to as ‘patient-controlled analgesia’ (PCA; or ‘patient-controlled analgesia therapy’). This empirical approach was not based on pharmacokinetics or on MEAC, but would provide a valuable complementary method for their investigation.

In the late 1960s, the concept of PCA with pethidine had been proposed for the alleviation of pain and for objective measurement of pain.^87^ By the late 1970s, PCA was progressively adopted into postsurgical patient management as new devices were developed.^88–90^ Some of the earliest PCA research studies in this period included measurements of concurrent plasma pethidine concentrations, and these confirmed the inter-patient variability in dosage requirements, pharmacokinetics and MEAC of pethidine.^91–93^ By the 1980s, developments in analytical chemistry methodology permitted reliable measurement of biofluid concentrations of most other opioid analgesic agents, thereby permitting analogous PCA research studies.

Concurrently, a variety of commercial programmable PCA devices were becoming available, and PCA had become a routine method for the management of pain. When reviewed in retrospect, studies performed under comparable conditions found only modest differences between their effectiveness, and had a high level of patient satisfaction, despite some common side effects such as nausea and vomiting.^94–96^ With pethidine, however, the occasional but unique problem of norpethidine-mediated CNS intoxication remained,^97–99^ and that would become a determining factor in its clinical demise in many countries.

## Pethidine: a new model analgesic agent

By the late 1970s, opioid receptors in the spinal cord had been described.^100,101^ This soon led to attempts to realise a role of neuraxial administration of the clinically available opioids for pain pharmacotherapy. The initial research was performed with intrathecally administered morphine.^102,103^ It seemed logical that epidurally administered pethidine might also be efficacious due to its greater lipophilicity and thus greater dural diffusion capacity than morphine. However, it was also recognised that any beneficial or adverse responses of epidurally administered pethidine could be complex due to its local anaesthetic activity and to centrally mediated effects from blood-borne drugs because of the larger doses expected to be required.^104,105^

Preliminary investigations with epidural pethidine in patients included concurrent measurements of reflex efferent sympathetic vasomotor activity and sudomotor activity to assess local anaesthetic activity. These demonstrated no impairment of efferent sympathetic activity and only small increases in resting blood flow in the treated area, from which it was concluded that pethidine-produced analgesia was not primarily mediated through its local anaesthetic activity.^106^ Ancillary studies included measurement of the time courses of blood and cerebrospinal fluid (CSF) pethidine concentrations after epidural administration in patients and after intravenous administration in dogs.^107^ After intravenous infusion, pethidine CSF concentrations were low, and similar to the unbound concentrations in blood.^105–107^ After epidural administration, the time course of analgesia corresponded with the time course of pethidine CSF concentrations rather than blood concentrations. Although blood-borne pethidine presumably contributes to the resultant analgesia, the combined observations supported the rationale that neuraxial pethidine acts principally by virtue of its spinal opioid receptor activity.^108^ Currently, many opioid analgesic agents are administered intrathecally and epidurally for treating postoperative and other types of pain, mostly successfully. Interestingly, the clinical utility of pethidine administered by neuraxial administration has maintained such a role,^108–112^ despite the demise of its systemic dosage forms.^113–116^

## Conclusion

In the 1930s, pethidine was found to coincidentally possess morphine-like analgesic and sedative properties. With widespread clinical use in the 1940s, it was found to be a versatile and effective substitute for morphine, with an antispasmodic action, but also addictive problems. In the 1970s, pethidine bioﬂuid concentrations could be measured reliably. This guided innovative forms of systemic and neuraxial dosing. In the 1990s, apart from neuraxial use, pethidine was becoming superseded. Pethidine, the ‘accidental’ opioid had served as a valuable ‘model’ opioid analgesic for over half a century.

## References

[1] Eisleb VO Schaumann O. Dolantin, ein neuartiges spasmolytikum und analgetikum (chemisches und pharmakologisches). Dtsch Med Wochenschr 1939; 65: 967–968. doi:10.1055/s-0028-1120563

[2] Michaelis N Schölkens B Rudolphi K . An anthology from Naunyn-Schmiedeberg’s archives of pharmacology. O. Schaumann (1940) Über eine neue Klasse von Verbindungen mit spasmolytischer und zentral analgetischer Wirkung unter besonderer Berücksichtigung des 1-methyl-4-phenyl-piperidin-4-carbonsäure-äthylesters (Dolantin). Archiv f. experiment. Path. u. Pharmakol. 196: 109–136. Naunyn-Schmiedeberg’s Arch Pharmacol 2007; 375: 81–84. doi:10.1007/s00210-007-0136-z 17310263

[3] Straub W. Eine empfindliche biologische Reaktion auf Morphin. Dtsch Med Wochenschr 1911; 37: 462.

[4] Eisleb O. Patent DE 679281C. Process for the preparation of piperidine compounds. Germany 1937. https://patents.google.com/patent/DE679281C/en?inventor=Dr+Otto+Eisleb&sort=old&page=1 (accessed 24 May 2025).

[5] Eisleb O. Patent US2167351A. Process for the preparation of piperidine compounds. USA 1938. https://patents.google.com/patent/US2167351A/en (accessed 24 May 2025).

[6] Schaumann O. Über eine neue Klasse von Verbindungen mit spasmolytischer und zentral analgetischer Wirkung unter besonderer Berücksichtigung des 1-methyl-4 phenyl-piperidin-4-carbonsäure-äthylesters (Dolantin). Archiv f experiment Path u Pharmakol 1940; 196: 109–136. doi:10.1007/bf01861099

[7] Schaumann O. Die neuen synthetischen Analgetika. Naunyn Schmiedebergs Arch Pharmakol 1951; 216: 48–77. doi:10.1007/bf00248060 12977259

[8] Schaumann O. Some new aspects of the action of morphine-like analgesics. Br Med J 1956; 2 1091–1093. doi:10.1136/bmj.2.5001.1091 PMC2035876 13364392

[9] Schäfer F. Schmerzbekämpfung in der Chirurgie mit Dolantin. Deutsch Med Wschr 1939; 65: 970–972. doi:10.1055/s-0028-1120564

[10] Duguid AME Heathcote RStA . Pharmacological action of ethylmethylphenylpiperidinecarboxylate. Quart J Pharm Pharmacol 1940; 13: 318–331.

[11] Batterman RC Himmelsbach CK. Demerol—a new synthetic analgesic: a review of its present status and comparison with morphine. J Amer Med Assoc 1943; 122: 222–226. doi:10.1097/00000542-194309000-00026

[12] Batterman RC. Clinical effectiveness and safety of a new synthetic analgesic drug, demerol. Arch Intl Med 1943; 71: 345–356. doi:10.1001/archinte.1943.00210030046004

[13] Weinstein ML. Demerol hydrochloride: a new drug in the practice of surgery. Amer J Surg 1943; 60: 267–269. doi:10.1016/s0002-9610(43)90663-4

[14] Hoffman R. Demerol, a new departure in analgesia: an evaluation of present therapeutic claims. Anesth Analg 1943: 22: 336–340. doi:10.1213/00000539-194301000-00050

[15] Hori CG Gold S. Demerol in surgery and obstetrics. Can Med Assoc J 1944; 51: 509–517. PMC1582053 20323279

[16] Walvood CA. Demerol: new substitute for morphine. MD Thesis, University of Nebraska Medical Center, USA 1944. https://digitalcommons.unmc.edu/mdtheses/1274 (Accessed 5 June 2025.)

[17] Kucher I. Zwei falle von dolantinsucht. Klin Wochenschr 1940; 19: 688–689.

[18] Schwarke R. Über Dolantinsucht. Dtsch Z Gesamte Gerichtl Med 1941; 35: 17–23.

[19] Himmelsbach CK. Further studies of the addiction liability of Demerol. J Pharmacol Exp Ther 1943; 79: 5–9. doi:10.1016/s0022-3565(25)09194-3

[20] Amending federal narcotic laws to apply to a recently discovered synthetic drug-(isonipecaine). 78th Congress, 2nd Session. Senate Report No.1004. June 21, 1944. Report. https://www.finance.senate.gov/download/1944/06/21/amending-federal-narcotic-laws-to-apply-to-a-recently-discovered-synthetic-drug-isonipecaine-report-78-1004&download=1 (accessed 5 June 2025).

[21] Way EL. Studies on the local anaesthetic properties of isonipecaine. J Am Pharm Assoc 1946; 35: 44–47. doi:10.1002/jps.3030350204 21018226

[22] Fussgānger R Schaumann O. Ůber ein neues Lokalanāsthetikum der Novokainreihe (Pantokain). Naunyn Schmiedebergs Arch Pharmakol Exp Pathol 1931; 160: 53–65. doi:10.1007/bf01863572

[23] Full page advertisement for pethidine in the Advertising Supplement of the Postgrad Med J, December 1944; i.

[24] Anon. A pain-killer. Milngavie and Bearsden Herald (Milngavie, Scotland), 20 March 1943, p.4.

[25] Anon. German drug kills pain – becomes habit. The Evening News (London, England), 26 October 1943, p.4.

[26] Anon. Ilicit traffic reported in morphine substitute. New York Herald Tribune (New York, NY, USA), 14 May 1944, p.8.

[27] Anon. Patients and pain. Smith’s Weekly (Sydney, Australia), 6 May 1944, p.17.

[28] Beckett AH Casy AF. Synthetic analgesics: stereochemical considerations. J Pharm Pharmacol 1954; 6: 986–1001. doi:10.1111/j.2042-7158.1954.tb11033.x 13212680

[29] Pert CB Snyder SH Portoghese PS. Correlation of opiate receptor affinity with analgetic effects of meperidine homologs. J Med Chem 1976; 19: 1248–1250. doi:10.1021/jm00232a015 11345

[30] Snyder SH Pasternak GW. Historical review: opioid receptors. Trends Pharmacol Sci 2003; 24: 198–205. doi:10.1016/s0165-6147(03)00066-x 12707007

[31] Jutkiewicz EM Traynor JR. Opioid analgesics. In: Brunton LL and Knollmann BC (eds) Goodman & Gilman’s: the pharmacological basis of therapeutics. 14th ed. New York: McGraw-Hill Education, 2025, pp.271–305.

[32] Wagner JG. History of pharmacokinetics. Pharmacol Ther 1981; 12: 537–562. doi:10.1016/0163-7258(81)90097-8 7025032

[33] Brodie BB Udenfriend S Dill W . The estimation of basic organic compounds in biological material. V. Estimation by salt formation with methyl orange. J Biol Chem 1947, 168: 335–339. 20291091

[34] Way EL Gimble AI McKelway WP , et al. The absorption, distribution and excretion of isonipecaine (Demerol). J Pharmacol Exp Therap 1949; 96: 477–484. doi:10.1016/s0022-3565(25)03707-3 18137903

[35] Burns JJ Berger BL Lief PA , et al. The physiological disposition and fate of meperidine (Demerol) in man and a method for its estimation in plasma. J Pharmacol Exp Therap 1955; 114: 289–296. 13243240

[36] Anders MW Mannering GJ. Application of gas chromatography to toxicology. Prog Chem Toxicol 1967; 3: 121–218. doi:10.1016/b978-1-4831-9988-7.50010-x 4868621

[37] Beckett AH Tucker GT Moffat AC. Routine detection and identification in urine of stimulants and other drugs, some of which may be used to modify performance in sport. J Pharm Pharmacol 1967; 19: 273–294. doi:10.1111/j.2042-7158.1967.tb08089.x 4382400

[38] Knowles JA White GR Ruelius HW. Determination of meperidine in human plasma and urine by gas-liquid chromatography. Analytical Letters 1973; 6: 281–290. doi:10.1080/00032717308062889

[39] Mather LE Tucker GT. Meperidine and other basic drugs: general method for their determination in plasma. J Pharm Sci 1974; 63: 306–307. doi:10.1002/jps.2600630232 4813264

[40] Chan K Kendall MJ Mitchard M. A quantitative gas-liquid chromatographic method for the determination of pethidine and its metabolites, norpethidine and pethidine N-oxide in human biological fluids. J Chromatogr A 1974; 89: 169–176. doi:10.1016/s0021-9673(01)99389-6

[41] Hartvig P Karlsson KE Lindberg C , et al. Simultaneous determination of therapeutic plasma concentrations of pethidine and norpethidine in man by electron capture gas chromatography. Eur J Clin Pharmacol 1977; 11: 65–69. doi:10.1007/bf00561790 832660

[42] Klotz U McHorse TS Wilkinson GR , et al. The effect of cirrhosis on the disposition and elimination of meperidine in man. Clin Pharmacol Ther 1974; 16: 667–675. doi:10.1002/cpt1974164667 4419525

[43] Mather LE Tucker GT Pflug AE , et al. Meperidine kinetics in man; intravenous injection in surgical patients and volunteers. Clin Pharmacol Ther 1975; 17: 21–30. doi:10.1002/cpt197517121 1091390

[44] Morgan D Moore G Thomas J , et al. Disposition of meperidine in pregnancy. Clin Pharmacol Ther 1978; 23: 288–295. doi:10.1002/cpt1978233288 627134

[45] Kuhnert BR Kuhnert PM Tu AS , et al. Meperidine and normeperidine levels following meperidine administration during labor: I. Mother. Am J Obstet Gynecol 1979; 133: 904–908. doi:10.1016/0002-9378(79)90310-7 434034

[46] Stambaugh JE Wainer IW. The bioavailability of meperidine. J Clin Pharmacol 1974; 14: 552–559. doi:10.1002/j.1552-4604.1974.tb01370.x 4430732

[47] Chan K Kendall MJ Mitchard M , et al. The effect of ageing on plasma pethidine concentration. Br J Clin Pharmacol 1975; 2: 297–302. doi:10.1111/j.1365-2125.1975.tb02773.x PMC1402607 1233988

[48] Mather LE Lindop MJ Tucker GT , et al. Pethidine revisited: plasma concentrations and effects after intramuscular injection. Br J Anaesth 1975; 47: 1269–1275. doi:10.1093/bja/47.12.1269 1218166

[49] Stambaugh JE Wainer IW Sanstead JK , et al. The clinical pharmacology of meperidine–comparison of routes of administration. J Clin Pharmacol 1976; 16: 245–256. doi:10.1002/j.1552-4604.1976.tb02401.x 1262534

[50] Mather LE Tucker GT. Systemic availability of orally administered meperidine. Clin Pharmacol Ther 1976; 20: 535–540. doi:10.1002/cpt1976205535 975724

[51] Morselli PL Rovei V. Placental transfer of pethidine and norpethidine and their pharmacokinetics in the newborn. Eur J Clin Pharmacol 1980; 18: 25–30. doi:10.1007/bf00561475 7398744

[52] Hull CJ. Compartmental models. Anaesth Pharmacol Rev 1994; 2: 188–203.

[53] Mather LE Runciman WB Ilsley AH , et al. A sheep preparation for studying interactions between blood flow and drug disposition. V. The effects of general and subarachnoid anaesthesia on blood flow and pethidine disposition. Br J Anaesth 1986; 58: 888–986. doi:10.1093/bja/58.8.888 3730236

[54] Runciman WB Myburgh J Upton RN , et al. Effects of anaesthesia on drug disposition. In: Feldman SA, Scurr CF, and Paton W (eds) Mechanisms of action of drugs in anaesthetic practice. 2nd ed. London: Edward Arnold, 1993, pp.83–128.

[55] Stambaugh JE Wainer IW. The bioavailability of meperidine using urine assays for meperidine and normeperidine. J Clin Pharmacol 1975; 15: 269–271. doi:10.1002/j.1552-4604.1975.tb01447.x 1092724

[56] Szeto HH Inturrisi CE. Simultaneous determination of meperidine and normeperidine in biofluids. J Chromatogr A 1976; 125: 503–510. doi:10.1016/s0021-9673(00)85713-1 993288

[57] Hartvig P Karlsson KE Lindberg C , et al. Simultaneous determination of therapeutic plasma concentrations of pethidine and norpethidine in man by electron capture gas chromatography. Eur J Clin Pharmacol 1977; 11: 65–69. doi:10.1007/bf00561790 832660

[58] Szeto HH Inturrisi CE Houde R , et al. Accumulation of normeperidine, an active metabolite of meperidine, in patients with renal failure or cancer. Ann Int Med 1977; 86: 738–741. doi:10.7326/0003-4819-86-6-738 869353

[59] Wainer IW Stambaugh JE. GLC determination of meperidinic and normeperidinic acids in urine. J Pharm Sci 1978; 67: 116–118. doi:10.1002/jps.2600670131 619098

[60] Szeto HH Clapp III JF Abrams R , et al. Brain uptake of meperidine in the fetal lamb. Am J Obstet Gynecol 1980; 138: 526–533. doi:10.1016/0002-9378(80)90281-1 7425023

[61] Trudnowski RJ Gessner T. Gastric excretion of intravenously administered meperidine in surgical patients. Anesth Analg 1979; 58: 88–92. doi:10.1213/00000539-197903000-00006 35041

[62] Mather LE Meffin PJ. Clinical pharmacokinetics [of] pethidine. Clin Pharmacokinet 1978; 3: 352–368. doi:10.2165/00003088-197803050-00002 359212

[63] Austin KL Stapleton JV Mather LE. Multiple intramuscular injections: a major source of variability in analgesic response to meperidine. Pain 1980; 8: 47–62. doi:10.1016/0304-3959(80)90089-5 7367036

[64] Austin KL Stapleton JV Mather LE. Relationship between blood meperidine concentrations and analgesic response. Anesthesiology 1980; 53: 460–466. doi:10.1097/00000542-198012000-00005 7457961

[65] Edwards D Svensson CK Visco JP , et al Clinical pharmacokinetics of pethidine: 1982. Clin Pharmacokinet 1982; 7: 421–433. doi:10.2165/00003088-198207050-00003 6754208

[66] Spector S. Quantitative determination of morphine in serum by radioimmunoassay. J Pharmacol Exp Ther 1971; 178: 253–258. doi:10.1016/s0022-3565(25)28956-x 4105659

[67] Spector S Vessell E. Disposition of morphine in man, Science 1971; 174: 421–422. doi:10.1126/science.174.4007.421 5111997

[68] Laitinen L Kanto J Vapaavuori M , et al. Morphine concentrations in plasma after intramuscular administration. Br J Anaesth 1975; 47: 1265–1267. doi:10.1093/bja/47.12.1265 1218165

[69] Garrett ER Gürkan T. Pharmacokinetics of morphine and its surrogates I: comparisons of sensitive assays of morphine in biological fluids and application to morphine pharmacokinetics in the dog. J Pharm Sci 1978; 67: 1512–1517. doi:10.1002/jps.2600671106 712585

[70] Stanski DR Paalzow L Edlund PO. Morphine pharmacokinetics: GLC assay versus radioimmunoassay. J Pharm Sci 1982; 71: 314–317. doi:10.1002/jps.2600710313 7069588

[71] Grabinski PY Kaiko RF Walsh TD , et al. Morphine radioimmunoassay specificity before and after extraction of plasma and cerebrospinal fluid. J Pharm Sci 1983; 72: 27–30. doi:10.1002/jps.2600720107 6827459

[72] Dahlström B Tamsen A Paalzow L , et al. Patient-controlled analgesic therapy, Part IV: pharmacokinetics and analgesic plasma concentrations of morphine. Clin Pharmacokin 1982; 7: 266–279. doi:10.2165/00003088-198207030-00006 7094501

[73] Milne RW Nation RL Somogyi AA. The disposition of morphine and its 3-and 6-glucuronide metabolites in humans and animals, and the importance of the metabolites to the pharmacological effects of morphine. Drug Metabolism Rev 1996; 28: 345–472. doi:10.3109/03602539608994011 8875123

[74] Faura CC Collins SL Moore RA , et al. Systematic review of factors affecting the ratios of morphine and its major metabolites. Pain 1998; 74: 43–53. doi:10.1016/s0304-3959(97)00142-5 9514559

[75] Glare PA Walsh TD. Clinical pharmacokinetics of morphine. Therap Drug Monit 1991; 13: 1–23. doi:10.1097/00007691-199101000-00001 2057987

[76] Wainer BH Wung WE Hill JH , et al. The production and characterization of antibodies reactive with meperidine. J Pharmacol Exp Ther 1976; 197: 734–743. doi:10.1016/s0022-3565(25)30562-8 945349

[77] Michiels M Hendriks R Heykants J. A sensitive radioimmunoassay for fentanyl: plasma level in dogs and man. Eur J Clin Pharmacol 1977; 12: 153–158. doi:10.1007/bf00645137 923629

[78] Cookson RF. Analgesic plasma concentrations. Br J Anaesth 1980; 52: 959. doi:10.1093/bja/52.9.959-a 7437232

[79] Prys-Roberts C. Pharmacokinetics of fentanyl. Anaesthesia 1986; 41: 428. doi:10.1111/j.1365-2044.1986.tb13233.x 3706691

[80] Fryirsa B Woodhouse A Huang JL , et al. Determination of subnanogram concentrations of fentanyl in plasma by gas chromatography—mass spectrometry: comparison with standard radioimmunoassay. J Chromatogr B 1997; 688: 79–85. doi:10.1016/s0378-4347(97)88058-9 9029316

[81] Mather LE. Trends in the pharmacology of opioids: implications for the pharmacotherapy of pain. Eur J Pain 2001; 5: 49–57. doi:10.1053/eujp.2001.0280 11798218

[82] Editorial. Postoperative pain. Br Med J 1978; 2: 517–518. doi:10.1136/bmj.2.6136.517 PMC1606999 698547

[83] Utting JE Smith JM. Post-operative analgesia. Anaesthesia 1979; 34: 320–332. doi:10.1111/j.1365-2044.1979.tb04929.x 36808

[84] Stapleton JV Austin K Mather LE. A pharmacokinetic approach to postoperative pain: continuous infusion of pethidine. Anaesth Intensive Care 1979; 7: 25–32. doi:10.1177/0310057x7900700103 434438

[85] Safe M. SA team smashes pain barrier. The News [Adelaide, South Australia], 8 November 1978, pp.1–3.

[86] Austin KL. Pharmacokinetic and pharmacodynamic studies of selected therapeutic agents. PhD Thesis, Flinders University of South Australia, 1980.

[87] Scott JS. Obstetric analgesia: a consideration of labor pain and a patient-controlled technique for its relief with meperidine. Amer J Obstet Gynecol 1970; 106: 959–978. doi:10.1016/s0002-9378(16)34084-4 5435661

[88] Hull CJ. Control of pain in the perioperative period. Br Med Bull 1988; 44: 341–356. doi:10.1093/oxfordjournals.bmb.a072255 3058258

[89] Owen H Mather LE Rowley K. The development and clinical use of patient-controlled analgesia. Anaesth Intensive Care 1988; 16: 437–447. doi:10.1177/0310057x8801600409 2906785

[90] White PF. Patient-controlled analgesia: an update on its use in the treatment of postoperative pain. Anesthesiol Clin North Am 1989; 7: 63–78. doi:10.1016/s0889-8537(21)00223-6

[91] Tamsen A Hartvig B Dahlstrom B , et al. Patient controlled analgesic therapy in the early postoperative period. Acta Anaesth Scand 1979; 23, 462–470. doi:10.1111/j.1399-6576.1979.tb01475.x 532541

[92] Tamsen A Hartvig P Fagerlund C , et al. Patient-controlled analgesic therapy, part I: pharmacokinetics of pethidine in the per-and postoperative periods. Clin Pharmacokinet 1982; 7: 149–163. doi:10.2165/00003088-198207020-00004 7067310

[93] Tamsen A Hartvig P Fagerlund C , et al. Patient-controlled analgesic therapy, part II: individual analgesic demand and analgesic plasma concentrations of pethidine in postoperative pain. Clin Pharmacokinet 1982; 7: 164–175. doi:10.2165/00003088-198207020-00005 7067311

[94] Hill HF Mather LE. Patient-controlled analgesia: pharmacokinetic and therapeutic considerations. Clin Pharmacokinet 1993; 24: 124–140. doi:10.2165/00003088-199324020-00003 8453822

[95] Woodhouse A Mather LE. Patient-controlled analgesia (PCA) for postoperative pain relief: what have we learned and where do we go from here? Analgesia 1997; 3: 1–14. doi:10.3727/107156997819566082

[96] Owen H Plummer J. Patient-controlled analgesia: current concepts in acute pain management. CNS Drugs 1997; 8: 203–218. doi:10.2165/00023210-199708030-00003

[97] Stone PA Macintyre PE Jarvis DA. Norpethidine toxicity and patient controlled analgesia. Br J Anaesth 1993; 71: 738–740. doi:10.1093/bja/71.5.738 8251291

[98] McHugh GJ. Norpethidine accumulation and generalized seizure during pethidine patient-controlled analgesia. Anaesth Intensive Care 1999; 27: 289–291. doi:10.1177/0310057x9902700419 10389564

[99] Plummer JL Gourlay GK Cherry DA. Norpethidine toxicity. Pain Reviews 2001; 8: 159–170. doi:10.1191/0968130201pr184ra

[100] Yaksh TL. Analgetic actions of intrathecal opiates in cat and primate. Brain Res 1978; 153: 205–210. doi:10.1016/0006-8993(78)91146-0 98219

[101] Snyder SH. Opiate receptors and internal opiates. Sci Am 1977; 236: 44–57. doi:10.1038/scientificamerican0377-44 190676

[102] Wang JK. Analgesic effect of intrathecally administered morphine. Reg Anesth Pain Med 1977; 2: 3–8.

[103] Behar M Olshwang D Magora F , et al. Epidural morphine in treatment of pain. Lancet 1979; ii: 527–529. doi:10.1016/s0140-6736(79)90947-4 85109

[104] Cousins MJ Mather LE Glynn CJ , et al. Selective spinal analgesia. Lancet 1979; i: 1141–1142. doi:10.1016/s0140-6736(79)91817-8 86861

[105] Glynn CJ Mather LE Cousins MJ , et al. Spinal narcotics and respiratory depression. Lancet i: 356–357, 1979. doi:10.1016/s0140-6736(79)90365-9 89412

[106] Glynn CJ Mather LE Cousins MJ , et al. Peridural meperidine in humans: analgesic response, pharmacokinetics, and transmission into CSF. Anesthesiology 1981; 55: 520–526. doi:10.1097/00000542-198111000-00007 7294405

[107] Mather LE Pavlin EG. Transfer of pethidine to CSF following intravenous administration. Anaesth Intensive Care 1981; 9: 205–207. doi:10.1177/0310057x8100900301 7283118

[108] Cousins MJ Mather LE. Intrathecal and epidural administration of opioids. Anesthesiology 1984; 61: 276–310. doi:10.1097/00000542-198409000-00008 6206753

[109] Ngan Kee WD . Intrathecal pethidine: pharmacology and clinical applications. Anaesth Intensive Care 1998; 26: 137–146. doi:10.1177/0310057x9802600202 9564390

[110] Ngan Kee WD . Epidural pethidine: pharmacology and clinical experience. Anaesth Intensive Care 1998; 26: 247–255. doi:10.1177/0310057x9802600303 9619217

[111] Rawal N. Intrathecal opioids for the management of post-operative pain. Best Pract Res Clin Anaesthesiol 2023; 37: 123–132. doi:10.1016/j.bpa.2023.01.001 37321761

[112] Bujedo BM Santos SG Azpiazu AU. A review of epidural and intrathecal opioids used in the management of postoperative pain. J Opioid Manag 2012; 8: 177–192. doi:10.5055/jom.2012.0114 22798178

[113] Pattullo G MacPherson R. Pethidine: the case for its withdrawal. In: Riley R (ed), Australasian anaesthesia. Melbourne, VIC: Australian and New Zealand College of Anaesthetists, 2011, pp.17–25.

[114] Harrison LR Arnet RE Ramos AS , et al. Pronounced declines in meperidine in the US: is the end imminent? Pharmacy 2022; 10: 154. doi:10.3390/pharmacy10060154 PMC9680368 36412830

[115] Boyle JM McCall KL Nichols SD , et al. Declines and pronounced regional disparities in meperidine use in the United States. Pharmacol Res Perspect 2021; 9; 1–6. doi:10.1002/prp2.809 PMC8204095 34128348

[116] Binder WJ Stearns JD Gorlin AW. Parenteral meperidine: a review of the pharmacology and clinical applications. Curr Anesthesiol Rep 2024; 14, 131–138. doi:10.1007/s40140-023-00597-7

